# Anti-IL-17 Antibody Improves Hepatic Steatosis by Suppressing Interleukin-17-Related Fatty Acid Synthesis and Metabolism

**DOI:** 10.1155/2013/253046

**Published:** 2013-12-17

**Authors:** Weidong Shi, Qiang Zhu, Jian Gu, Xiaoshan Liu, Ling Lu, Xiaofeng Qian, Jian Shen, Feng Zhang, Guoqiang Li

**Affiliations:** ^1^Translational Medicine Research Center, Jiangning Hospital Affiliated to Nanjing Medical University, Nanjing 211100, China; ^2^Key Laboratory of Living Donor Liver Transplantation of Ministry of Public Health, Nanjing 210029, China; ^3^General Surgery, Second People's Hospital of Nantong, Nantong 226002, China; ^4^Nanjing Children's Hospital Affiliated to Nanjing Medical University, Nanjing 210008, China; ^5^Department of General Surgery, Second Affiliated Hospital of Nanjing Medical University, Nanjing 210000, China

## Abstract

To investigate the relationship between interleukin-17 and proteins involved in fatty acid metabolism with respect to alcoholic liver disease, male ICR mice were randomized into five groups: control, alcoholic liver disease (ALD) at 4 weeks, 8 weeks, and 12 weeks, and anti-IL-17 antibody treated ALD. A proteomic approach was adopted to investigate changes in liver proteins between control and ALD groups. The proteomic analysis was performed by two-dimensional difference gel electrophoresis. Spots of interest were subsequently subjected to nanospray ionization tandem mass spectrometry (MS/MS) for protein identification. Additionally, expression levels of selected proteins were confirmed by western blot. Transcriptional levels of some selected proteins were determined by RT-PCR. Expression levels of 95 protein spots changed significantly (ratio >1.5, *P* < 0.05) during the development of ALD. Sterol regulatory element-binding protein-lc (SREBP-1c), carbohydrate response element binding protein (ChREBP), enoyl-coenzyme A hydratase (ECHS1), and peroxisome proliferator-activated receptor alpha (PPAR-**α**) were identified by MS/MS among the proteins shown to vary the most; increased IL-17 elevated the transcription of SREBP-1c and ChREBP but suppressed ECHS1 and PPAR-**α**. The interleukin-17 signaling pathway is involved in ALD development; anti-IL-17 antibody improved hepatic steatosis by suppressing interleukin-17-related fatty acid metabolism.

## 1. Introduction 

Alcoholic liver disease is becoming more and more widespread but the mechanisms that underlie the condition remain unknown. The typical characteristics of alcoholic liver disease are hepatic steatosis (otherwise known as fatty liver), hepatitis, fibrosis, and cirrhosis. Hospital mortality rates for alcoholic hepatitis can be as high as 60%, and mortality is often due to failure and cirrhosis of the liver [1].

Complex immune responses play an important role in the development of alcoholic liver disease [2]. Chronic long-term intake of alcohol impairs the protective mechanisms of the gut; this results in increased serum levels of lipopolysaccharide (LPS) in the area of the portal vein, which in turn activates Kupffer cells. The activated Kupffer cells secrete inflammatory cytokines, such as tumor necrosis factor *α* (TNF-*α*), which initiate a cascade of inflammatory events that can lead to inflammation.

T helper 17 (TH17) cells are a newly discovered subset of T helper cells that are independent of the traditional lineages of TH1 and TH2 cells. TH17 cells appear to be involved in many autoimmune diseases [3]. Cytokine IL-17 is secreted mainly by TH17 cells and has comprehensive biological functions. The liver tissue of patients with alcoholic liver disease contains a large number of cells that secrete IL-17, and the degree of injury shows a positive correlation with the number of IL-17-positive cells that have infiltrated into the liver [4].

Hepatic steatosis is characterized by the accumulation of triglycerides in the liver. Further studies are required to determine whether IL-17 promotes hepatic steatosis by interfering with fatty acid synthesis and metabolism.

The purpose of this study was to explore the relationship between IL-17 and proteins involved in fatty acid synthesis and metabolism and to search for new targets for pharmacotherapy to treat hepatic steatosis.

## 2. Material and Methods

### 2.1. Sample Collection and Preparation

Male mice of the strain ICR (8 weeks of age; 18–25 g; obtained from the Animal Center of Nanjing Medical University) were housed under controlled environmental conditions with respect to temperature (20–22°C), light (12-h light/12-h dark cycle), and humidity (50–70%), and received food and water ad libitum in accordance with the National Research Council's guide (Permit number 17-0956) for the care and use of laboratory animals. In this study we used a flexible and stable mouse model of alcoholic liver disease that represented an improvement on the model of Tsukamoto-French [5]. The male mice were divided randomly into five groups: control (*n* = 6), ALD (4W) (*n* = 8), ALD (8W) (*n* = 12), ALD (12W) (*n* = 14), and Anti-IL-17 antibody treated ALD (*n* = 6; this group was equivalent to the ALD (12W) group but the animals were treated with Anti-IL-17 antibody for the 8th of the experimental period). All mice were sacrificed after 12 h of fasting. Blood samples were collected for biochemical assays. The liver was removed, rinsed with ice-cold saline, and weighed. It was then frozen immediately in liquid nitrogen and stored at −80°C until further analysis or fixed in formalin and embedded in paraffin for evaluation by hematoxylin and eosin (HE) staining.

To analyse the effect of IL-17 in vivo, 8-to 12-week-old male ICR mice were injected intravenously with 1 *μ*g of IL-17 dissolved in saline. The mice were then sacrificed and the livers were collected 16 hours later, control mice were injected with saline only.

### 2.2. Analysis of Liver Enzymes

Serum aspartate aminotransferase (AST) and alanine aminotransferase (ALT) levels were measured by using an assay kit (Transaminase C, WAKO, Osaka, Japan). Levels of Gamma-glutamyltransferase (GGT), alkaline phosphatase (ALK-P), and total bilirubin were assayed using an automatic biochemical analyzer.

### 2.3. Cytokine Determination by ELISA

Serum IL-17 levels were determined by using a mouse enzyme-linked immunosorbent assay (ELISA) kit (Biosource, San Jose, CA, USA). Analyses were performed in accordance with the manufacturer's instructions.

The blood samples were collected After 6 h of fasting because the cytokine levels increased more rapidly than the transaminase levels and returned to almost normal levels within 12 h.

### 2.4. Flow Cytometry Analysis

Thl7 cells accounted for the proportion of mononuclear cell detection in liver tissue. Take some fresh liver tissue and grind it after cuting it to pieces, make it into suspension cells. Cells were resuspened in 5% fetal bovine serum RPMI1640 and filted through a 200 mesh metal mesh filter. After that, lymphocyte isolation liquid (Ficoll) was used to obtain mononuclear cells. Cell survival rate was determined by trypan blue with the survival rate of (98 + 3.2)%. Mononuclear cells were stained with FITC rat anti-mouse—CD 4 and APC rat anti-mouse-IL-17 antibody (BD Pharmingen, San Diego,CA, USA). Result were detected through flow cytometry, negative tube was used as control.

### 2.5. Two-Dimensional Difference Gel Electrophoresis (2-DE) and Protein Identification by MALDI-TOF/TOF

Each sample containing 120 *μ*g proteins was loaded in IPG strips (24 cm, pH 3–10, NL, GE Healthcare, San Francisco, CA, USA) for rehydration. Following isoelectric focusing, the IPG strips were equilibrated to maintain the fully reduced state of the proteins, and to prevent the reoxidation of thiol groups during electrophoresis. Second-dimensional electrophoresis was performed on 12.5% SDS gels in an Ettan-DALT-six electrophoresis unit (GE Healthcare, San Francisco, CA, USA) and gels were visualized by silver staining as described previously. In this experiment, data were generated from three independently run gels at each group (12 gels in total). The stained gels were scanned, and the ImageMasterTM 2-D Platinum Software (Version 5.0, Amersham Bioscience, Swiss Institute of Bioinformatics, Geneva, Switzerland) was used for spot detection, quantification, and comparative analyses. We averaged the values from the three independent samples, respectively, calculated the means and standard deviations, and assessed statistical significance with Student's *t*-tests using ImageMasterTM 2D platinum software. *P* values less than 0.05 were considered statistically significant.

The differentially expressed protein spots were then excised and identified. Briefly, the protein spots were dehydrated in acetonitrile (ACN), reduced using 10 mM DTT/25 mM NH4HCO3 at 56°C for 1 h, and subsequently alkylated with 55 mM iodoacetamide/25 mM NH4HCO3 in the dark at room temperature for 45 min. Gel fragments were thoroughly washed with 25 mM NH4HCO3, 50% ACN, and 100% ACN and dried in a SpeedVac. Dried gel fragments were reswollen with 2–3 *μ*L trypsin solution (Promega, Madison, WI, USA) (10 ng/*μ*L in 25 mM NH4HCO3) at 4°C for 30 min. Excess liquid was discarded and the gel plugs were incubated at 37°C for 12 h. Trifluoroacetic acid (TFA) at a final concentration of 0.1% was added to stop the digestive reaction. The digests were immediately spotted onto 600 *μ*m AnchorChips (Bruker Daltonics, Bremen, Germany). Spotting was achieved by pipetting 1 *μ*L of the analyte onto the MALDI target plate in duplicate and subsequently adding 0.05 *μ*L of 2 mg/mL *α*-HCCA in 0.1% TFA/33% acetonitrile containing 2 mM (NH4)3PO4. All samples were analyzed on a time-of-flight Ultraflex II mass spectrometer (Bruker Daltonics) set to the positive-ion reflectron mode.

Each acquired mass spectrum (m/z range, 700–4000; resolution, 15000–20000) was processed using the FlexAnalysis v2.4 and Biotools 3.0 (Bruker Daltonics) software packages with the following specifications: peak detection algorithm: Sort Neaten Assign and Place (SNAP); S/N threshold: 3; and quality factor threshold: 50. Trypsic autodigestion ion picks (842.51, 1045.56, 2211.10, and 2225.12 Da) were used as internal standards to validate the external calibration procedure. Matrix and/or autoproteolytic trypsin fragments were removed. The masses of the peptides obtained were cross-referenced with the NCBI mouse database with the use of Mascot (v2.1.03) in an automated mode that used the following search parameters: a significant protein score at *P* < 0.05; minimum mass accuracy: 100 ppm; trypsin as the enzyme; one missed cleavage sites allowed; cysteine carbamidomethylation, acrylamide modified cysteine, methionine oxidation and similarity of pI, and the relative molecular mass specified, with the minimum sequence coverage at 15%. Protein identification was confirmed by sequence information automatically obtained from the MS/MS analysis. Acquired MS/MS spectra were also processed using the software FlexAnalysisTM 2.4 using a SNAP method set at a signal-to-noise ratio threshold of 3.0. The MS/MS spectra were automatically searched in the NCBI mouse database by Mascot (v2.4). Search parameters for MS/MS data were set to 100 ppm for the precursor ion and 0.3 Da for the fragment ions. Cleavage specificity and covalent modifications were considered, as described above. The score was higher than the minimum significant individual ion score (*P* < 0.05). All significant MS/MS identifications by Mascot were manually verified for spectral quality and matching y and b ion series. When multiple entries corresponded to slightly different sequences, only the database entry that exhibited the highest number of matching peptides was included.

### 2.6. Western Blotting

Protein levels were determined by western blotting. The amount of protein was 20 *μ*g. After the protein had been transferred to polyvinylidene fluoride membranes, the membranes were blocked in phosphate buffered saline (PBS) with 0.1% Tween, 5% (w/v) skim milk powder, and 10% horse serum for 2 hours and then incubated with the appropriate specific antibody (anti-sterol regulatory element binding protein-lc (SREBP-1c) antibody, 1 : 300; anti-carbohydrate response element binding protein (ChREBP) antibody, 1 : 150; anti-enoyl-coenzyme A hydratase (ECHS1) antibody, 1 : 300; anti-peroxisome proliferator-activated receptor alpha (PPAR-*α*) antibody, 1 : 100) overnight at 4°C. The membranes were then incubated in a 1 : 2500 dilution of horseradish peroxidase-conjugated secondary antibody (Santa Cruz Biotechnology Inc., Santa Cruz, CA, USA) at 37°C for 1 h. The results were visualized by using a chemiluminescence detection system (Pierce ECL Western Blotting Substrate, Thermo Scientific), followed by exposure to autoradiography film (Kodak Biomax XAR film). The expression in each sample was analyzed with Quantity One 4.4.0 software (Bio-Rad Laboratories, Hercules, CA, USA).

### 2.7. Cell Culture and Transient Transfection Assays

Human L02 hepatocytes and AML-12 cells (acquired from ATCC Rockefeller, MD, USA) were grown in Dulbecco's modified Eagle's medium (Cambrex, Verviers, Belgium) containing 10% fetal bovine serum (FBS) and antibiotics. Cells were treated with 5, 10, or 25 ng/mL IL-17 for 24 hours before reverse-transcription polymerase chain reaction (RT-PCR) analysis.

### 2.8. Fluorescence Quantitative RT-PCR Analysis

Primer Premier 5.0 software was used to design appropriate primers in accordance with the manufacturer's instructions. Total RNA was extracted from liver tissue with Trizol reagent (Gibco, Carlsbad, CA, USA) and used to prepare cDNA by reverse transcription. Aliquots of 0.4 *μ*L of cDNA template were added to each 25 *μ*L reaction mix. After labeling, each tube was placed in quantitative fluorescence detector (Rotor-Gene 3000) for PCR amplification. The concentration of each transcript of interest, together with that of *β*-actin, was calculated directly by the machine. The concentration of each transcript was normalized by dividing it by the concentration of *β*-actin to give the relative amount of transcript.

### 2.9. Statistical Analysis

Data are expressed as the mean ± standard error (SE). Analysis of variance was used to compare the means of three groups, followed by Newman-Keuls test to determine the statistical significance between two groups. *P* < 0.05 was considered to be statistically significant.

## 3. Results 

### 3.1. Hepatic Histology

**Figure 1:**
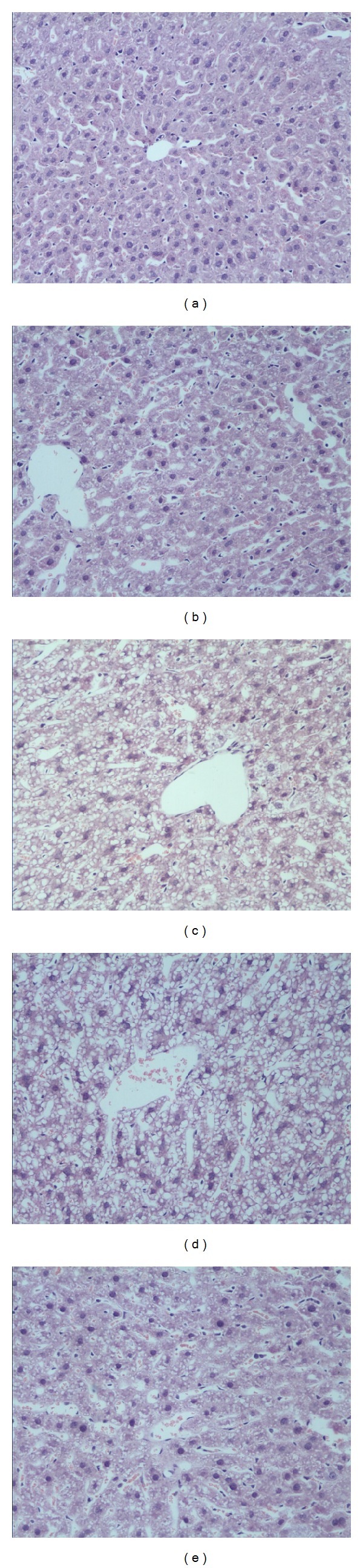
Hepatic pathology. Liver sections stained with hematoxylin and eosin from mice. (a) Control group, (b) ALD (4W) group, (c) ALD (8W) group, (d) ALD (12W) group, and (e) Anti-IL-17 antibody treated ALD group. Original magnification ×200. Data are representative of three separate experiments.

Liver tissue from all groups was stained with HE (Figure 1). Typical steatosis was observed in the ALD (8, 12W) group as compared with the other groups. Some slightly form of Hepatic Hepatitis was observed in the ALD (12W) group. The form of steatosis was obviously ameliorated after treatment with Antibody-IL-17 from the 8th week.

### 3.2. Biochemical Indicators

**Figure 2:**
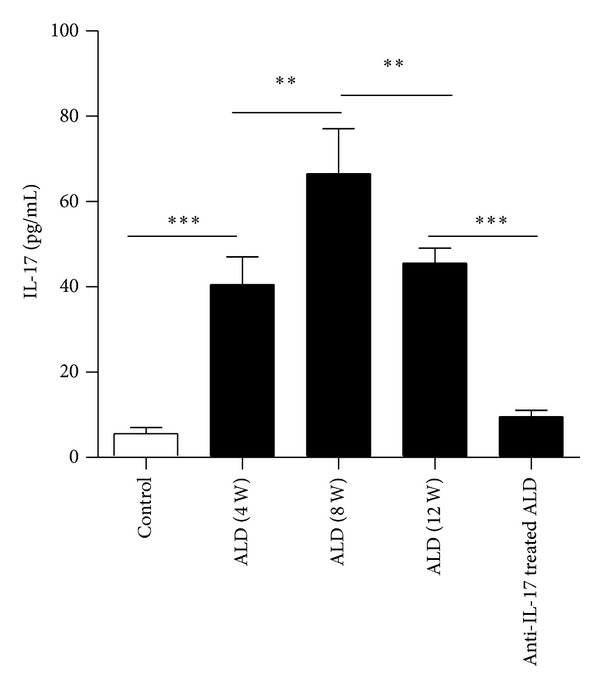
Serum IL-17 levels as determined by ELISA in the different stages of ALD. (a) Control group, (b) ALD (4W) group, (c) ALD (8W) group, (d) ALD (12W) group, and (e) Anti-IL-17 antibody treated ALD group. The data indicate Mean ± SEM of three separate experiments. **P < 0.01; ***P < 0.001.

Serum ALT, AST, and GGT levels were highest in the ALD (12W) group as compared with the other groups. All the ALD groups had higher serum ALT, AST, and GGT levels than the control group (Data not shown). In the process of establishing the mouse model, we discovered that the serum IL-17 level was highest in the 8th month followed by the 12th month (Figure 2).

### 3.3. The Level of IL-17 in Liver Tissue of Every ALD Group

**Figure 3:**
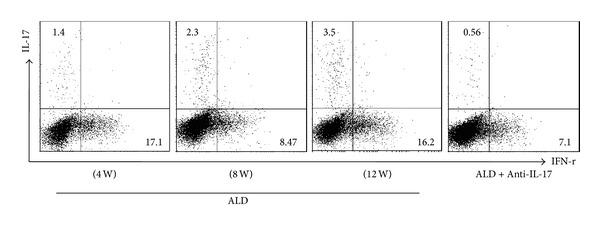
Activation of a peripheral IL-17-secreting phenotype in liver tissue of every ALD group. Data are representative of three separate experiments.

To determine whether T lymphocytes from ALD groups might contribute to high circulating IL-17 level, we analyzed their PBMCs by flow cytometry (Figure 3). The proportions of IL-17+ cells in every group are (1.4)%, (2.3)%, (3.5)%, and (0.56)%, respectively.

### 3.4. Quantitative Proteomic Analysis

**Figure 4:**
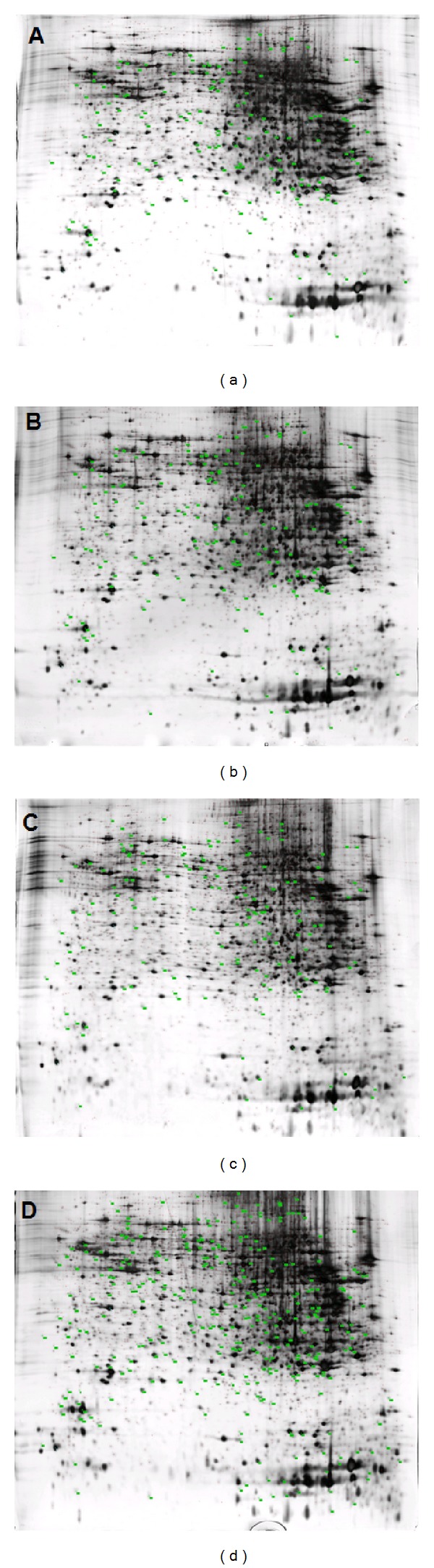
Differentially expressed protein spots displayed in 2DE images. Representative 2DE images from: (a) the ALD (4W) group, (b) the ALD (8W) group, (c) the ALD (12W) group, and (d) Anti-IL-17 antibody treated ALD group. Data are representative of three separate experiments.

**Figure 5:**
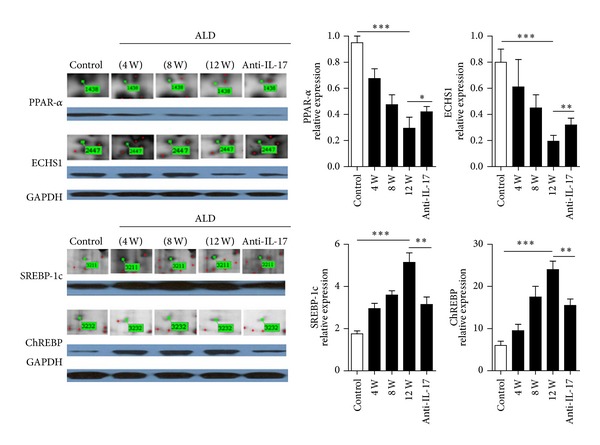
Western blot validation of four selected proteins. Hepatic expression levels of sterol regulatory element-binding protein-lc (SREBP-1c), carbohydrate response element binding protein (ChREBP), enoyl-coenzyme A hydratase (ECHS1), and peroxisome proliferator-activated receptor alpha (PPAR-*α*) in the ALD and control groups. The results correlated well with the quantification of the DIGE images, which is also shown. The data indicate Mean ± SEM of five separate experiments. *P < 0.05; **P < 0.01; ***P < 0.001.

To identify protein molecules that were interesting with respect to alcoholic liver disease, we compared protein expression in the ALD and control groups by means of 2D-PAGE (Figure 4). On the basis of the statistical analysis, 95 protein spots changed significantly between the control and ALD groups. Among them, the expression of 35 proteins changed more than 1.5-fold, and six of these proteins showed more than a twofold change in abundance. These protein spots were characterized by tandem mass spectrometry (MS/MS). Among the 35 protein spots analyzed, 14 contained more than one protein; these spots were considered not to have been clearly identified and were excluded from further analysis. Six spots could not be identified at all. The remaining 15 proteins were identified unambiguously. Among them, proteins 1438, 2447, 3211, and 3232 were identified as SREBP-1c, ChREBP, ECHS1, and PPAR-*α*, which are key proteins in fatty acid synthesis and metabolism. When the ALD groups were compared with the control group, the expression levels of SREBP-1c and ChREBP were elevated whereas the levels of ECHS1 and PPAR-*α* were reduced. The results of western blotting were in accordance with those of the quantitative proteomic analysis (Figure 5).

### 3.5. Fluorescence Quantitative RT-PCR Analysis

**Figure 6:**
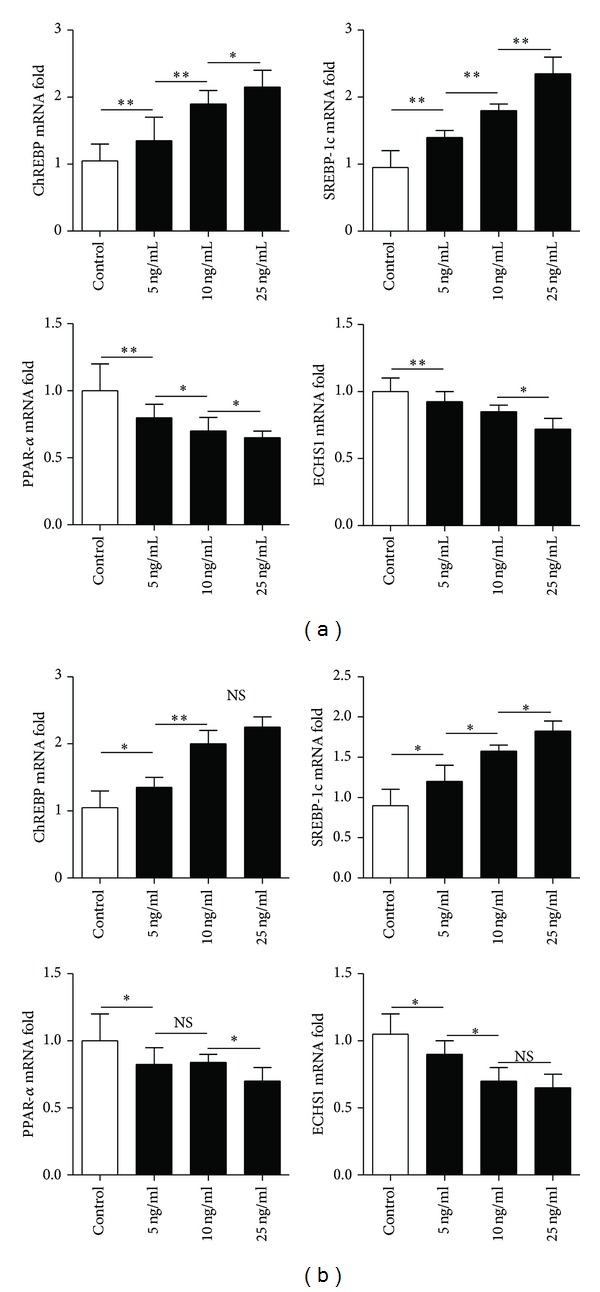
The effect of different serum levels of IL-17 in L02 cells (a) or in AML-12 cells (b) on the transcript levels for the four selected proteins. Gene expression levels in mice from the control group were set at 1. The data indicate Mean ± SEM of five separate experiments. *P < 0.05; **P < 0.01; NS: no significance.

**Figure 7:**
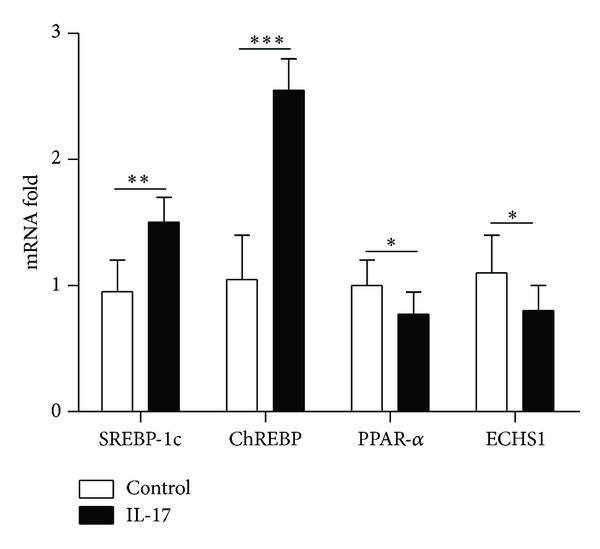
The effect of an intravenous dose of 1 *μ*g of IL-17 on the transcript levels for the four selected proteins in vivo. The expression levels of SREBP-1c, ChREBP, ECHS1, and PPAR-*α* mRNA were determined in livers collected 16 hours after being intravenously injected with IL-17. Gene expression levels in mice from the control group were set at 1. The data indicate Mean ± SEM of three separate experiments. *P < 0.05; **P < 0.01; ***P < 0.001.

The effect of increased levels of IL-17 on the transcription of the genes for the above mentioned proteins was examined in vivo and in vitro. The results of the quantitative RT-PCR analysis showed that, both in vivo and in vitro, the transcription of SREBP-1c and ChREBP was elevated, whereas the transcription of ECHS1 and PPAR-*α* was suppressed as the level of IL-17 was increased (Figures 6 and 7).

## 4. Discussion 

Imbalances of both the immune system and metabolism are involved in the development of alcohol-induced liver injury. However, so far, the underlying mechanisms have not been elucidated completely. The innate immune response and the adaptive immune response both participate in the etiology of alcoholic liver disease [2]. The innate immune system includes macrophages (Kupffer cells), dendritic cells (DC) cells, natural killer (NK) cells, natural killer T (NKT) cells, inflammatory cytokines, acute phase response proteins, and chemokines [6, 7]. The adaptive immune response, especially the autoimmune response, increases the severity of alcohol-induced liver injury.

IL-17, a more recently discovered cytokine, has a wide range of biological functions, such as the recruitment of neutrophils and the release of other inflammatory factors. These effects are mediated by IL-17 receptors, which are expressed in multiple tissues: vascular endothelial cells, peripheral T cells, and B- cell lineages, together with fibroblast, lung, myelomonocytic, and marrow stromal cells [8–11]. It has been reported that large numbers of TH17 cells and high levels of serum IL-17 are found in many autoimmune diseases, including multiple sclerosis (MS), inflammatory bowel diseases, rheumatoid arthritis, Lyme disease, contact dermatitis, psoriasis, uveitis, and experimental autoimmune encephalomyelitis (EAE) [12–14]. IL-17 is involved in the development of alcoholic liver disease [8]. Our findings revealed that serum IL-17 levels increased progressively with increasing time in the mouse model of alcoholic liver disease and the serum levels of ALT, AST, and GGT also increased in parallelism.

In this study, we systematically analyzed the proteome during different stages of alcoholic liver disease in our mouse model, which might help to elucidate the mechanisms that are involved in the progression of the disease. We identified 95 protein spots that changed significantly between the control and ALD groups. By using MS/MS analysis, four of the proteins were identified as SREBP-1c, ChREBP, ECHS1, and PPAR-*α*.

SREBPs are transcription factors of the basic-helix-loop-helix leucine zipper (bHLH-Zip) family and bind to the DNA sequence TCACNCCAC, which constitutes the sterol regulatory element. SREBPs are encoded by the genes SREBP1 and SREBP2 [15]. Expression of the SREBP-1 gene produces two different protein isoforms, SREBP-1a and -1c. SREBP-1c is responsible for regulating the genes required for de novo lipogenesis [16].

ChREBP is a recently described transcription factor that also belongs to the bHLH-Zip family [17] and regulates carbohydrate metabolism in the liver [18]. Increased expression of ChREBP increases the activity of lipase, which leads to a high level of fatty acids in the liver [19].

ECHS1 functions in the second step of the mitochondrial fatty acid *β*-oxidation pathway of fatty acid metabolism. It catalyzes the hydration of 2-trans-enoyl-coenzyme A (CoA) intermediates to L-3-hydroxyacyl-CoAs. Oxidation of fatty acids occurs in the subcellular organelles, with *β*-oxidation confined to the mitochondria and peroxisomes and *ω*-oxidation occurring in the endoplasmic reticulum [20, 21]. Impairment of the function of ECHS1 inhibits the *β*-oxidation of fatty acids in the mitochondria and can account for the storage of excess lipid in the liver [22–24]. PPAR-*α* belongs to the peroxisome proliferator-activated receptor superfamily and regulates the transcription of many genes that encode enzymes involved in fatty acid *β*-oxidation.

It was of interest to determine whether the levels of these four proteins were correlated tightly with the serum IL-17 level. We found that as the level of IL-17 was increased, the transcription of SREBP-1c and ChREBP was also increased, whereas the transcription of ECHS1 and PPAR-*α* was suppressed, both in vivo and in vitro. Allicin improved the degree of hepatic steatosis by reducing the level of IL-17 and thus affecting IL-17-related fatty acid synthesis and metabolism.

## 5. Conclusion

Our study indicated that the interleukin-17 signaling pathway is involved in the development of ALD. Increased IL-17 elevated the transcription of SREBP-1c and ChREBP but suppressed ECHS1 and PPAR-*α*; anti-IL-17 antibody improved hepatic steatosis by suppressing interleukin-17-related fatty acid metabolism.
